# Diagnostic double strike in the emergency room - two cases of complete pancreatic ruptures due to bicycle handlebar injuries on two consecutive days

**DOI:** 10.1186/s13256-018-1594-2

**Published:** 2018-03-26

**Authors:** A. M. Luu, K. Meurer, T. Herzog, W. Uhl, C. Braumann

**Affiliations:** Department of General and Visceral Surgery, St. Josef-Hospital, Ruhr - University Bochum, Gudrunstr. 56, 44791 Bochum, Germany

**Keywords:** Pancreatic rupture, Bike fall, Bicycle handlebar injury, Pancreatic fracture, Blunt abdominal trauma

## Abstract

**Background:**

Pancreatic injuries are rare in cases of blunt abdominal trauma and therefore easily misdiagnosed at time of hospital admission. They are associated with a significantly elevated morbidity and lethality. Bicycle handlebar injuries are the most common cause of pancreatic trauma in children and adolescents.

**Case presentation:**

We report two cases of a 23-year-old Caucasian woman and a 15-year-old Caucasian boy who presented to our clinic with a similar history of a bicycle accident on 2 consecutive days. Both suffered from a fall from a bicycle with bicycle handlebar injury 4 and 6 days prior to admission in our clinic. Emergency distal pancreatectomies were performed in both cases.

**Conclusions:**

Pancreatic injuries must be highly suspected in bicycle handlebar injuries, even if amylase/lipase levels or ultrasound findings seem unremarkable. The best initial strategies are early computed tomography and a quick referral to a level 1 trauma center. Distal pancreatectomy is the treatment of choice in cases of complete rupture of the pancreatic body.

## Background

Pancreatic injuries are present in 0.6% of abdominal traumas in children [1]. They occur rarely because the pancreas is located in the retroperitoneum where it is relatively protected by the surrounding tissues [2]. Bicycle injuries are the most common cause of pancreatic injuries in children [3].

We report the cases of two patients who presented to our clinic 2 days in a row with a similar history of a bicycle accident and complete pancreatic rupture. These cases are presented to emphasize the importance of an early complete diagnostic workup to evaluate the severity of a blunt abdominal trauma. They were unusual due to the similar history of a misdiagnosed and underestimated life-threatening trauma as well as a referral to our clinic 2 days in a row.

## Case presentation

### Case 1

**Fig. 1:**
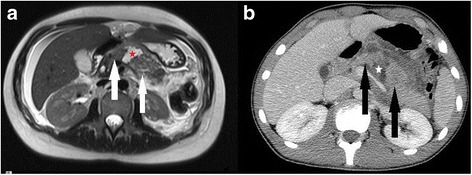
T2-weighted magnetic resonance imaging (**a**) of patient 1 and computed tomography (**b**) of patient 2 depicting complete rupture of the pancreas (*white* and *black arrows*). Fluid collection between the ruptured pancreas (*star*)

**Fig. 2:**
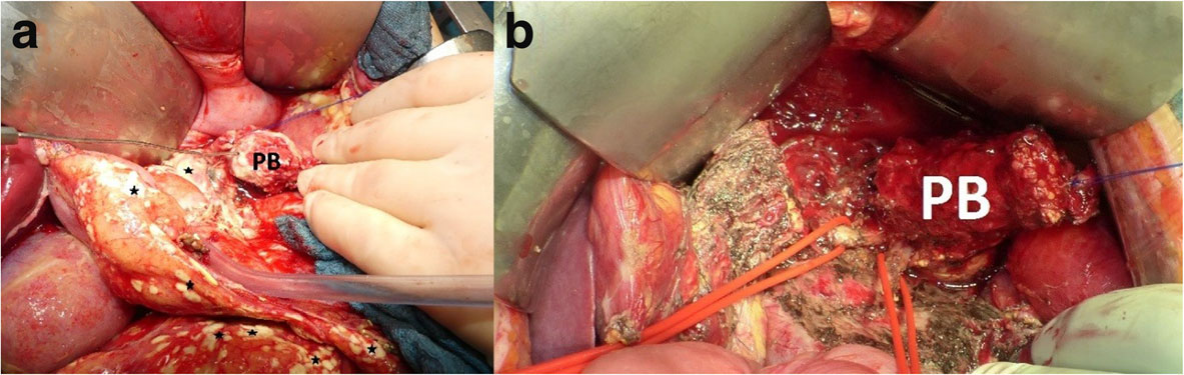
Intraoperative view of complete rupture of the pancreas of patients 1 and 2. **a** A testing probe is inserted in the main pancreatic duct of patient 1 indicating the ruptured pancreatic body. Multiple calcifications indicate locally advanced peritonitis and necrotizing pancreatitis (*stars*). Right loop (**b**): splenic vein. Central loop (**b**): inferior mesenteric vein. *PB* pancreatic body

A 23-year-old Caucasian woman suffered from blunt abdominal trauma due to a fall from a bicycle onto the bicycle handlebar. She was a university student and had an unremarkable medical history. Social history was significant for occasional tobacco smoking and alcohol consumption. She was treated in a Dutch hospital for 6 days prior to transfer to our clinic. Magnetic resonance imaging (MRI) revealed complete rupture of the pancreatic body (Fig. 1): grade 3 pancreatic trauma (see Table 1) [4]. Unsuccessful stenting of the ruptured main pancreatic duct (MPD) was performed via endoscopic retrograde cholangiopancreatography (ERCP). Computed tomography (CT)-guided drainages were placed percutaneously in epigastric fluid collections. She became septic and was then transferred to our clinic with the following medication: intravenously administered metamizole 500 mg (every 6 hours), subcutaneous octreotide 100 μg (1 dose in the morning, 1 dose at midday, and 1 dose in the evening), intravenously administered pantoprazole 40 mg (1 dose in the morning, 0 dose at midday, and 0 dose in the evening), and intravenously administered ceftriaxone 2 g (1 dose in the morning, 0 dose at midday, and 0 dose in the evening). On admission, she was hemodynamically unstable (blood pressure 85/65 mmHg, heart rate 120/minute, oxygen saturation 95%, and body temperature 37.5 °C) with a significantly increased C-reactive protein level of 212 mg/l (< 5) and procalcitonin level of 10.7 ng/ml (< 0.005). The following laboratory values were determined as well: White blood cell count 8830/μl (4600–9500), hemoglobin 9.8 g/dl (12–16), thrombocytes 225,000/μl (150,000–400,000), international normalized ratio (INR) 1.13 (0.8–1.1), partial thromboplastin time 30 seconds (26–40), amylase 169 U/l (13–53), lipase 183 U/l (13–60), lactate dehydrogenase 219 U/l (135–214), aspartate amino transferase 21 U/l (10–35), alanine amino transferase 14 U/l (10–35), cholinesterase 2404 U/l (4260–11,250), gamma glutamyl transferase 42 U/l (6–42), alkaline phosphatase 56 U/l (35–104), bilirubin 0.4 mg/dl (< 1.2), and creatinine 0.41 mg/dl (0.5–0.9). Blood cultures were positive for *Pseudomonas aeruginosa*. General abdominal tenderness was palpable indicating peritonitis. Her cardiorespiratory state was remarkable for tachycardia and hypotension. A neurological examination was unremarkable apart from a somnolent state. A transverse explorative laparotomy was performed immediately. On intraoperative examination, a severe peritonitis and necrotizing pancreatitis were identified as complications of a complete rupture of the pancreatic body (Fig. 2). Furthermore, the percutaneous drainage had perforated her transverse colon. A distal pancreatectomy, splenectomy, removal of the drainage, suturing of the colon perforation, and a protective ileostomy were performed. Subsequently, she underwent two further surgeries with abdominal cavity lavages. Posttraumatic complications in this case comprised severe peritonitis, necrotizing pancreatitis, critical illness polyneuropathy, and pancreatic fistula. She spent 48 days in our surgical intensive care unit and was discharged on postoperative day 68.

**Table 1:** Pancreas Organ Injury Scale of the American Association for the Surgery of Trauma [4]

Grade	Injury	Description
1	Hematoma/Laceration	Minor contusion or superficial laceration without duct injury
2	Hematoma/Laceration	Major contusion or laceration without duct injury or tissue loss
3	Laceration	Distal transection or parenchymal injury with duct injury
4	Laceration	Proximal transection or parenchymal injury involving ampulla
5	Laceration	Massive disruption of pancreatic head

A follow-up investigation 6 months after the primary surgery showed a regular postoperative state without signs of pancreatitis, fluid collections, or abscesses. Reconstruction of the ileostomy was uneventful.

### Case 2

A 15-year-old Caucasian boy was transferred to our clinic exactly 1 day after case 1. He was a middle school student and had an unremarkable medical history. His social history was unremarkable for tobacco smoking, alcohol, or drugs. Four days before, he fell from a bicycle and hit his abdomen on the bicycle handlebar just like the first patient. Initial ultrasound and laboratory tests were uneventful. However, his amylase and lipase levels were not tested. CT was performed due to progressive abdominal pain which again showed a complete rupture of the pancreatic body; once again grade 3 pancreatic trauma [4]. Laboratory values were significant for C-reactive protein 66 mg/l (< 5), amylase 160 U/l (13–53), and lipase 150 U/l (13–60). The following laboratory values were determined as well: White blood cell count 9650/μl (4600–9500), hemoglobin 12.4 g/dl (14–18), thrombocytes 176,000/μl (150,000–400,000), INR 1.16 (0.8–1.1), partial thromboplastin time 37 seconds (26–40), lactate dehydrogenase 223 U/l (< 271), aspartate amino transferase 16 U/l (< 46), alanine amino transferase 15 U/l (< 45), cholinesterase 5131 U/l (5320–12,920), gamma glutamyl transferase 17 U/l (< 29), alkaline phosphatase 116 U/l (< 381), bilirubin 0.9 mg/dl (< 1.2), and creatinine 0.56 (0.8–1.4). He was then transferred to our hospital with the following medication: acetaminophen 500 mg (1 dose in the morning, 1 dose at midday, and 1 dose in the evening) and pantoprazole 40 mg (1 dose in the morning, 0 dose at midday, and 0 dose in the evening). The following vital parameters were taken on admission: blood pressure 100/80 mmHg, heart rate 98/minute, oxygen saturation 100%, and body temperature 37.9 °C. A physical examination revealed a general tenderness of his abdomen indicating peritonitis. Neurological and cardiorespiratory examinations were unremarkable. An explorative laparotomy with subsequent distal pancreatectomy and splenectomy was performed. Lower grade signs of peritonitis were present intraoperatively compared to case 1 (Fig. 2). Postoperatively, he developed a pancreatic fistula. He was discharged on postoperative day 29.

A follow-up investigation 6 months after the primary surgery revealed a regular postoperative state without signs for infections, fluid collections, or abscesses.

Both patients were vaccinated against *Streptococcus pneumoniae*, *Haemophilus influenza*, and *Neisseria meningitidis* 6 weeks after discharge to prevent overwhelming postsplenectomy infection syndrome.

## Discussion

We experienced in 2 consecutive days two rare cases of complete pancreatic rupture after bicycle handlebar injuries with significant diagnostic delay. Both patients suffered from trauma a couple of days before admission to our clinic and where treated in other hospitals first. Stenting of the MPD via ERCP was unsuccessful in patient 1 due to complete transection of her pancreas. It is of note that pancreatic injuries involving ruptures of the MPD must undergo surgical treatment [5]. In patient 2 amylase levels were not tested during admission in another hospital. Initial abdominal ultrasound was unremarkable. A CT scan was performed afterwards due to progressive abdominal pain which showed the complete transection of his pancreas. Diagnostic delay does easily occur in cases of blunt abdominal trauma, due to underestimation of the traumatic extent. Symptom-free and silent intervals can simulate an assumed recovery [6, 7]. Both our patients underwent emergency distal pancreatectomy and required a prolonged stay in our intensive care unit due to sepsis and peritonitis. The first patient suffered from a more severe peritonitis and critical illness polyneuropathy. Our cases confirm that delayed surgical treatment of pancreatic ruptures is associated with an increased morbidity [8].

Pancreatic injuries including complete rupture of the MPD due to blunt abdominal trauma are rare. Signs and symptoms can be minimal impeding early and correct diagnosis. Typical mechanisms of the accident are related to falls from bicycles onto the bicycle handlebars [3] or motor vehicle accidents with sudden deceleration by seatbelt straps [2]. While ultrasound of the abdomen is usually performed first, CT, magnetic resonance cholangiopancreatography (MRCP), and ERCP are thought to be the most valuable diagnostic techniques to identify a complicated pancreatic trauma with injury of the MPD [9]. Laboratory values are significant for elevated serum amylase in 90% of cases [3]. However, in children the rise in amylase levels can be delayed for more than 12 hours after the trauma [10, 11]. Non-operative strategies apply in up to 76% of cases and are common in lower grade injuries [1, 9, 11]. Higher grade injuries involving rupture of the MPD are present in 10–24% of cases. They are usually treated surgically [1, 3, 9]. Emergency surgery is required in cases of peritonitis, large amounts of free intraabdominal fluid, evidence of MPD rupture, and complete transection of the pancreas [5]. Morbidity rate can exceed 60% while the in-hospital mortality rate is 5% [1, 12]. Common complications comprise pancreatitis, peritonitis, abscess formations, pseudocysts, and splenic artery aneurysms [11, 12].

Regular follow-up investigations are required 3, 9, and 15 months after surgery and include physical examination, ultrasound, and CT or MRI.

Our cases show the importance of a timely diagnosed pancreatic trauma. Diagnostic delay increases the morbidity and can have fatal consequences. Single curative treatment in complete ruptures of the pancreatic body is a distal pancreatectomy. Factors aggravating the morbidity include young age, severity of the injury, amylase levels, and duration of shock [2, 13].

## Conclusions

Surgeons should be aware of pancreatic trauma especially in blunt abdominal accidents involving bicycle handlebar injuries even in cases of normal serum amylase levels or initially unremarkable ultrasound findings. A complete diagnostic workup requires repeat physical examination, sequential amylase/lipase tests, sonography, early CT of the abdomen or MRI, as well as a quick referral to a level 1 trauma center. While lower grade pancreatic injuries can respond to conservative management, complete pancreatic ruptures must be treated surgically.
